# Dynamic and Systemic Perspective in Autism Spectrum Disorders: A Change of Gaze in Research Opens to A New Landscape of Needs and Solutions

**DOI:** 10.3390/brainsci12020250

**Published:** 2022-02-11

**Authors:** Cristina Panisi, Marina Marini

**Affiliations:** 1 Fondazione Istituto Sacra Famiglia ONLUS, Cesano Boscone, 20090 Milan, Italy; 2 Department of Experimental, Diagnostic and Specialty Medicine, School of Medicine, University of Bologna, 40126 Bologna, Italy; marina.marini@unibo.it

**Keywords:** autism, prevention, epigenetics, neuroinflammation, microbiota, mitochondrial impairment, oxidative stress, healthcare, multi-disciplinarity, comorbidities

## Abstract

The first step for a harmonious bio-psycho-social framework in approaching autism spectrum disorders (ASD) is overcoming the conflict between the biological and the psychosocial perspective. Biological research can provide clues for a correct approach to clinical practice, assuming that it would lead to the conceptualization of a pathogenetic paradigm able to account for epidemiologic and clinical findings. The upward trajectory in ASD prevalence and the systemic involvement of other organs besides the brain suggest that the epigenetic paradigm is the most plausible one. The embryo-fetal period is the crucial window of opportunity for keeping neurodevelopment on the right tracks, suggesting that women’s health in pregnancy should be a priority. Maladaptive molecular pathways beginning in utero, in particular, a vicious circle between the immune response, oxidative stress/mitochondrial dysfunction, and dysbiosis-impact neurodevelopment and brain functioning across the lifespan and are the basis for progressive multisystemic disorders that account for the substantial health loss and the increased mortality in ASD. Therefore, the biological complexity of ASD and its implications for health requires the enhancement of clinical skills on these topics, to achieve an effective multi-disciplinary healthcare model. Well-balanced training courses could be a promising starting point to make a change.

## 1. Introduction

Autism is an early onset, and in most cases lifelong, neurodevelopmental condition, currently diagnosed on the basis of the clinical assessment of behavioral features [1]. The wide range of different behavioral characteristics and the degree of intellectual impairment account for the wide “spectrum” of clinical pictures and their varied impacts on major life areas [2] and quality of life [3]. This wide clinical heterogeneity highlights the need to provide personalized answers to complex and unique needs, including the empowerment and participation of individuals with ASD and their families in society and in decisions concerning them [4,5].

The consistent upward trajectory in autism prevalence since the 1990s [6] is cause of concern. Current prevalence is estimated as 1:54 in the USA [7] and 1:77 in Italy [8]. The broadening of the diagnostic criteria certainly contributes to explaining the upward trend [8], however, converging evidence suggests the role of numerous environmental factors [9,10,11,12].

The frequent medical comorbidities [13] go hand-in-hand with the manifold metabolic, immunologic, and microbial imbalances, integrating the clinical picture and suggesting that ASD is a multidimensional, systemic, and evolving condition [14], currently not entirely accounted by diagnostic criteria that are limited to the assessment of behavioral characteristics.

With this review we wish to draw the attention of clinicians to the multidimensional, personalized, and integrated health care model required to meet the complex health needs of this broad, and increasing, psycho-neurobiological condition and to the need to pay particular attention to the period that precedes the clinical diagnosis, including the embryo-fetal window, since the crucial events for neurodevelopment occur before the clinic onset of ASD. To that end, we are going to pinpoint a number of key issues, integrating our previous review [14] with information that we deem useful to this purpose.

We will first address the recent increase in prevalence of ASD that should be understood within the broader model of an ongoing epidemiological transition, affecting also non-communicable diseases other than neurodevelopmental disorders. The awareness of the underlying reasons for the recent increase in prevalence of ASD may help to design and analyze cost-effective models in healthcare and to suggest effective strategies to make a change.

Then, we will summarize the main biological evidence underlying the complexity of the pathogenetic model of ASD, detailing the broad array of metabolic, immunologic, and microbiological imbalances affecting ASD people, from pregnancy throughout their entire lifespan. We will point out how biological characteristics should be harmonically integrated in the bio-psycho-social framework [15,16], overcoming the persistent conflicting attitude between the biological perspective and the psycho-social approach. Such faulty approach prevents research from achieving the degree of complexity required for a multidimensional integration of the body-mind-society connections and cause services and clinical practice to miss the opportunity of a complete multidimensional assessment and intervention, including an accurate evaluation of medical comorbidities.

Finally, we will point out the urgency to provide early and integrated support to children at high-risk of neurodevelopmental disorders, so as not to lose the opportunity of exploiting early-life neuroplasticity, in particular, in the time window that precedes the diagnosis (*First 1000 Days* [17]). The lack of support in the first 2 years of life, the delay in diagnosis, and the latency between diagnosis and the beginning of the psycho-educational interventions impact the severity of the impairment, affecting the individual quality of life and increasing the social burden. Therefore, best efforts should be done to bridge the gap and strengthen the support to children and families in the period of maximum neuroplasticity.

In the following paragraphs, some biological issues, not limited to ASD research, are discussed, with the purpose of highlighting the connections between some topics rather than describing them in detail. These will constitute the premises for understanding the shortcomings of current clinical practice and propose coherent strategies to ameliorate both research and clinical intervention.

## 2. The Biological Basis of Autism Spectrum Disorders

### 2.1. Genetics in ASD

The great clinical heterogeneity of ASD persons may imply a great heterogeneity in the pathogenesis of ASD. Besides a ~10% incidence of monogenic syndromic mutations [18], more than 200 susceptibility genes have been identified to be associated with autism until now [19], although none of the individual genes identified to date accounts for more than 1% of ASD cases [20]. The remaining ~90% of ASD cases (so-called “idiopathic autism”) are ascribed to gene variations which have been defined as “common polygenic risk” [21], and include thousands of loci with copy number variations (CNV) and single nucleotide polymorphisms (SNP), whether de novo or inherited, often determining a multifactorial unique and synergic gene asset. In any case, the genetic architecture of the autistic trait is extremely complex. For instance, the concordance rate in monozygotic twins was found to be highly variable (36–96%), depending on sex, on trait severity, and on whether the diagnosis was narrowly or broadly defined [22,23,24,25]. In addition, a recent analysis [25] concluded “although ASD itself is highly heritable, variation-in-severity of symptomatology above the diagnostic threshold is substantially influenced, in contrast, by non-shared environmental factors which may identify novel targets of early ASD amelioration”.

#### More Relevant ASD Susceptibility Genes

By examining the functions of proteins encoded by ASD susceptibility genes, three main functional categories emerge [18,21]. They include: (i) proteins involved in cell-to-cell signaling, along cell membranes and especially at the synapses (these include in particular the synaptic gene transcription and translation pathway, neural cell adhesion molecules, scaffolding proteins in synapse, ion channel proteins, neuron transmitter reporters), (ii) proteins involved in signal transduction and in protein–protein interactions, and (iii) DNA- and RNA-binding and chromatin-modifying proteins. Most strikingly, these three functional categories interact within regulatory networks at multiple levels along neuronal biological processes, so that the dysregulation of one protein affects the neural cell as a whole [18,21].

Several ASD syndromic mutations involve pathways that anchor synaptic machinery, regulate synaptic vesicle release, control subcellular signaling pathways, and orchestrate neuronal migration and connections [26]. The finding that the same molecular functions, cellular locations, and processes are shared by gene products coded by genes mutated in rare monogenic mutations leading to ASD [27] has led to the captivating definition of ASD as “synaptopathy” [28]. Axonal projection, dendritic formation and arborization, maintenance and elimination of synapses are all critical steps in neural differentiation [29], and abnormalities in synapse formation and function cause imbalance between neuronal excitation and inhibition [30]. However, different syndromic, as well as different rare monogenic disorders, affect proteins with functional roles other than those related to synaptic functions, e.g., those involved in the epigenetic control of gene expression. Undoubtedly, the definition of ASD as synaptopathy is fascinating but rather reductive.

### 2.2. Non-Linear Epigenetics and Paradigm Shift for Understanding Neurodevelopmental Disorders

The incomplete concordance between homozygous twins mentioned above [25], especially if penetration is taken into consideration, is per se a proof that environmental factors play a role in the ASD phenotype. Needless to say, these factors exert themselves through epigenetic mechanisms. Epigenetics refers to gene regulatory mechanisms independent of alterations of the underlying DNA coding sequences [31]. Epigenetic effectors, i.e., DNA methylation, histone tails modifications, and non-coding RNAs [32], influence gene transcription patterns through multiple mechanisms, regulating the accessibility of genomic loci to several regulatory factors (i.e., enhancers, silencers, transcription factors). During ontogenesis, changes in epigenetic signatures regulate the differentiation of precursor cells into their specific mature state [32], contributing to the embryo-fetal development, which defines the morphological and functional differentiation of the different brain areas, but also prepares embryo-fetal tissues and organs to face the extra-uterine environment through the tuning of metabolic-immuno-neuro-endocrine pathways [31,33,34]. During embryo-fetal programming, reactive-adaptive-predictive epigenetic modifications tune gene expression in response to the continuously and rapidly changing environment (fetal programming) [35].

Epigenetic mechanisms are also the most plausible explanation for the increase in prevalence of ASD in the past decades [12,14] since the hypothesis of an increasing number of monogenic mutations is not realistic. Therefore, non-linear epigenetics, rather than linear genetics, seems to be a plausible explanation for such complex conditions [36].

The supposed reasons for the steep increase in prevalence of neurodevelopmental disorders as a whole are the same for the increased prevalence of numerous inflammatory, metabolic, degenerative, and neoplastic diseases [35], as put forward by the Developmental Origin of Health and Diseases Theory (*DOHaD Theory*) which suggests an epigenetic/embryo-fetal origin of diseases [37,38]. According to the DOHaD paradigm, most non-communicable conditions originate during early development as the result of environmental influences: altered nutrition, stress, drugs, infections or exposure to environmental chemicals [17,37]. During pregnancy, multiple homeostatic mechanisms support the development of the growing fetus. In the case of prenatal adversities, optimal homeostatic equilibrium might be impaired and maladaptive responses might initiate pathogenic mechanisms, with long-term health consequences [39]. Heindel and Vandenberg (2015) [38] report that 343 published epidemiology studies (through mid-2013) suggested associations between developmental chemical exposures and later life diseases, specifically including, but not limited to, neurobehavioral and/or learning disabilities or IQ loss. Several animal studies [40] and recent human studies [41] have supported this view. The epigenetic paradigm appears to be the most plausible molecular model that links genes, environment, and the susceptibility to disease. Moreover, one of the most impressive epigenetic mechanisms, the transmission of disease risk across generations [42], has been described in relation to carcinogen exposure during a prenatal susceptibility window [43]. The *First 1000 Days*, from conception to age 2 [17], are considered to be the most critical period for neuro-development, which makes this temporal window the most vulnerable one to mechanisms interfering with the epigenetic machinery. In this period, several exogenous insults changing the maternal milieu and the exposure to adverse environmental events cause long-term modifications in epigenetic patterns, which display a high level of plasticity [44] during the embryo-fetal development. With regards to neurodevelopment, these affect neurogenesis, cell migration, differentiation, and synaptogenesis [45]. Placental physiology and placental gene expression are affected by several maternal factors, such as over and under-nutrition, drug and alcohol intake, smoking, a wide range of environmental toxicants, infections and stress [46,47]. Worth noting, a large array of environmental pollutants is activated by the aryl hydrocarbon receptor (AhR)/cytochrome P450 (CYPs) pathway, which in turn affects the epigenetic modulation of ASD-relevant genes [48]. Abruzzo et al. (2019) reported a significant increase of the AhR signaling pathway in sera of ASD children [49]. Emberti-Gialloreti et al. (2019) reviewed evidence concerning numerous risk and protective factors in pregnancy that are correlated with ASD in the offspring [50].

The three most common approaches to DNA methylation pattern profiling are global, epigenome-wide (EWAS), and candidate-gene DNA methylation analyses [51]. Dall’Aglio et al. (2018) recently reviewed epigenetic markers in ASD [51]. Of the 24 selected studies, the majority focused on DNA methylation and had a cross-sectional design. The most commonly examined tissues were blood and cerebellum. The majority of the reviewed publications used a candidate gene or EWAS approach to test the presence of differential epigenetic marks. In the proximity of three candidate genes, differential DNA methylation was demonstrated [51]. Histone modification changes were significant in the gene coding for H3K27 in the cerebellum and cortex of autistic people compared to control [51]. These findings suggest a causal association between epigenetic modifications and neurodevelopmental disorders, although some results were either conflicting or not independently replicated, requiring further insights [51]. Undoubtedly, future research will be able to clarify these discrepancies.

Maternal immune response is one of the effector arms of epigenetic dysregulation [52]. Notably, maternal immune activation (MIA) leads to the remodeling of some chromosomal regions and leads to changes in gene expression in the brain of the offspring [53]. A multidirectional interaction between maternal immune response during pregnancy and epigenetic machinery regulates ontogenesis and neurodevelopment. Imbalances in this interplay may disturb fetal brain wiring and start processes with long-term consequences [52]. Mechanisms include immune factor-induced changes in epigenetic signatures in the brain, dysregulation of epigenetic modifications specifically in genomic regions that encode immune functions, and aberrant epigenetic regulation of microglia. [52]. The manifold interplay between fetal epigenetic machinery and maternal immune response draws attention to maternal immune balance during pregnancy and to the broad array of factors that influence it. In fact, MIA is an effector pathway triggered by several risk factors, including infections, autoimmunity, allergy, maternal-fetal stress, environmental toxicants [54]. We are obliged to underline that the aspects associated with the immune response have already been dealt with in a previous review which we co-authored [14]; consequently, we will summarize below only some essential points, while we will introduce new data and new considerations that we believe useful for framing these important pathogenetic aspects.

### 2.3. The Multifaceted Immune System along the Way of Neurodevelopment

The immune system deeply affects brain development, both in physiological and in abnormal trajectories [55,56,57]. All the steps of neurodevelopment, i.e., neuronal migration, synaptogenesis, white matter organization, and remodeling (pruning), are influenced by the immune response [56]. Kipnis (2018) calls the immune system the “seventh” sense, highlighting the constructive role of immune information in the wiring of the connectome, being “the five senses” the common ones (visual, olfactory, gustatory, somatosensory, and auditory) and the sixth sense the proprioceptive system and the vagal nerve [58]. Kipnis poses that the early optimal multimodal integration is the premise for body awareness and, hence, the physiological neurodevelopmental trajectory [58]. Maternal cytokines and chemokines are involved in migration of neuronal precursors, neuronal maintenance, synaptic pruning, and neuroplasticity [59]. Abnormal immune activation may therefore impair these roles [58,60]. On one side, growing evidence shows that maternal immune activation during pregnancy impacts on the neurodevelopmental trajectory [61,62,63], on the other, numerous abnormalities in immune pathways involved in embryo-fetal neurodevelopment show correlation to ASD (reviewed by Sotgiu et al., 2020 [64]). MIA can be considered as a “primer condition” and a predisposing frailty background. Besides the heightened risk of ASD in childhood, MIA offspring seem to be more susceptible to “second hits” induced by stress and drug abuse during adolescence and have heightened risk of psychiatric and neurologic disorders in adulthood [60]. This statement is supported by animal models and clinical studies concerning MIA, showing neurodevelopmental, neuropsychiatric, and neurologic disorders as a result of a trajectory of potential frailty, rather than static images of inevitable and irreversible diseases written in genes [60,65].

A broad variety of maternal immune mechanisms are involved in MIA, both in specific and in natural immunity. As for the former, maternal autoantibodies targeted against the Folate Receptor α (FRα) provide one of the clearest examples to understand the relevance of biological issues in research and clinical practice in ASD. Folate is one of the vitamins of the B-complex. It plays a pivotal role in neurodevelopment during the ontogenesis and in the first years of life [66]. The concentration of folate in the developing brain relies on active import mechanisms—based on FRα—to cross both placental and brain barriers, and anti-FRα autoantibodies (FRAAs) inhibit this transport [67]. The correlation between maternal FRAAs and ASD-like features in the offspring has been demonstrated in animal models [67] and is consistent with the positive correlation between the severity of ASD symptoms in autistic children and the presence of FRAAs in their mothers [66]. The high prevalence, much higher than in controls, of FRAAs in 58–76% of children with ASD and the correlation between the blood titers of FRAAs with folate levels in the cerebrospinal fluid further stress the relevance of this mechanism [68,69]. A systematic review and meta-analysis found that treatment with d,l-leucovorin (folinic acid) is associated with improvements in core symptoms and other behavior abnormalities [70]. Maternal FRAAs are correlated with sterility and poor outcomes of pregnancy [71]. Cross-reactivity with cow milk FR may be a plausible explanation of the production of FRAA. In fact, downregulation of FR autoimmunity in cerebral folate deficiency syndrome was reported after adopting milk-free diet [72]. The increased gut permeability in pregnancy [73] is a risk factor for autoimmunity [74]. Taken as a whole, these apparently independent events allow us to speculate that maternal FRAA may represent a crucial effector arm in the context of MIA, highlighting pathogenic mechanisms that can be targeted in the prevention. In fact, these findings strengthen the impact of immune response on neurodevelopment, underline the pivotal role of the folate metabolism, suggest FRAAs as useful biomarkers [70], and provide evidence for preventive and therapeutic opportunities [68,70].

A pivotal role in MIA is played by IL-6, a major pro-inflammatory cytokine [60]. As a consequence of the increase in the placental levels of IL-6, placental production of insulin-like growth factor 1 (IGF-1) is decreased [75]. IGF-1 is a pivotal factor for fetal growth and development. In utero growth retardation (IUGR) caused by impaired uteroplacental blood supply (utero-placental IUGR) is correlated with lower fetal IGF-1 gene expression in experimental animals and circulating IGF-1 in human fetuses [76]. IGF-1 controls the glucose metabolism in the developing brain. It has significant effects on fetal and perinatal brain growth—especially in neurogenesis, synaptogenesis, and myelination—and participates in several physiologically relevant neuroprotective mechanisms [77]. Oligodendrocytes, cerebellar Purkinje cells, and motor neurons seem to be particularly sensitive to IGF-1 deficiency [78]. Overall, IGF-1 in normal fetal and neonatal nerve development prevents neuronal injury, reduces neuronal degeneration, and increases myelination [78]. Depressed neonatal serum IGF-1 is a predictor of reduced or delayed central nervous system (CNS) development, especially in preterm infants. Strong correlations have been found between low neonatal serum concentrations of IGF-1 and poor brain and retinal growth as well as poor general growth with multiorgan morbidities [79]. These findings might be relevant to ASD and explain differences in brain connectivity, since autistic children under four years of age show lower concentrations of IGF-1 than age-matched controls [80]. Therefore, alterations of the IGF-1 pathway in brain development during pregnancy and in the early post-natal period provide a plausible explanation that links MIA with the pathophysiology of the placental insufficiency and numerous risk factors that are correlated to ASD [81]. These findings are consistent with the higher risk of neurodevelopmental disorders in preterm infants and the 7- to 10-fold increased risk of ASD when compared to term born infants [82]. The semantic map proposed by Grossi et at. (2018) effectively represents these connections [83].

Garbett KA et al. (2012) explored the mechanisms by which maternal infection increases the risk for schizophrenia and autism in the offspring by administering three MIA-inducing treatments (influenza virus, poly(I:C) and interleukin IL-6) to mouse dams and examining the immediate effects on the fetal brain transcriptome [84]. All three MIA treatments led to strong and common gene expression changes in the embryonic brain, which suggest that the response to MIA might be a neuroprotective attempt carried out by the developing brain to counteract environmental stress. The cost of this reaction is the disruption of typical neuronal differentiation and axonal growth. The authors conclude that this cascade of events might parallel the mechanisms by which environmental insults contribute to the risk of neurodevelopmental and neuropsychiatric disorders such as ASD and schizophrenia [84].

Among infectious agents, major concerns relate to the current SARS-CoV-2 pandemic. In fact, while the transplacental transmission of this virus is unlikely to occur [85], maternal infections may impact fetal neurodevelopment through the MIA. Placentas from infected women are more likely to show aspects of maternal and/or fetal malperfusion as compared to healthy controls; fibrin deposition and intense recruitment of inflammatory infiltrates are the most common findings [86]. Therefore, impaired uteroplacental blood supply and a maternal cytokine storm might interfere with fetal epigenetic machinery [87]. Within the cytokine imbalance, IL-6 is of particular interest both for the aforesaid role played in MIA, including the link with IGF-I, and for the correlation with adverse clinical outcomes of COVID-19 [88,89]. Maternal stress is a further risk factor related to COVID-19 pandemic [90], with complex relationships within the multifaceted bio-psycho-social framework [91]. Therefore, the inflammatory environment and the risk of impairment in the placental perfusion in mothers with COVID-19 during pregnancy suggest an increased risk of abnormal neurodevelopment, which should be ascertained in the next few years [87].

Discussion of the immune response in pregnancy includes the maternal microbiota, a complex system that is constantly evolving during pregnancy and adapting to the energetic fetal needs [92,93]. The maternal microbiota may impact the fetus via maternal factors (i.e., maternal immune responses and microbial metabolites that cross the placenta [94,95], or via factors that may mediate epigenetic programming in the fetus, such as diet [96], or maternal stress [97], which also affect the maternal microbiota.

The topics and links which have been described in this paragraph show a cross-section of the many possible paths that neurodevelopment can take and how this complex process cannot be considered an automatic execution of instructions written in DNA.

Numerous risk and protective factors have been linked to the onset of ASD in the offspring, highlighting the need of paying particular attention to maternal diet, nutraceutical supplementation, prevention and treatment of metabolic abnormalities, protection from toxicant exposure, and numerous other factors linked with an increased risk for ASD [92]. However, single factors can rarely be considered causal. Rather, as stated above, each factor contributes to frailty trajectories. Therefore, evaluating single factor exposures separately through traditional linear regression models seems to be an oversimplification. On the contrary, the evaluation of synergistic effects of different simultaneous exposures seems to be closer to the biologic condition that is object of study [98]. The Auto Contractive Map Artificial Neural Network (ANN) suggested by Grossi et al. (2018) highlights the hidden trends and associations among multiple variables related to pregnancy and reveals the risk profiles related to ASD [83]. The study provides relevant suggestions both for research and clinical practice. The application of machine learning systems and the building of ever-increasing databases which might be continuously fed by clinical, laboratory, and instrumental records—represent the most promising strategy to track, starting from the womb, different risk profiles in the lifelong neuropsychiatric trajectory. Through the reproduction of the dynamical interaction of multiple factors simultaneously, this tool is able to represent problems of high complexity.

## 3. The Web of Metabolic, Immunologic, and Microbiologic Imbalance in ASD People

The link between immune response and neurodevelopment begins in the womb and continues throughout life. Interest for neuroinflammation in ASD began with the demonstration of cerebral inflammation in autoptic brains of autistic people [99]. Numerous studies, reviewed by Matta et al. (2019) [100], demonstrate reactive microglia and astrocytes, altered glial structure and function, cytokine profiles, and gut immune dysfunction in ASD people and animal models, providing strong evidence for the interactions between the nervous system and immune pathways [100].

The immunologic imbalance in ASD concerns both innate immunity and the specific response of T lymphocytes, with a shift toward a Th1-type pattern and prevalence of pro-inflammatory cytokines, increase of B-lymphocytes, NK cells, and dendritic cells; and different expression of surface markers [101]. Abruzzo et al. (2019) found a correlation between clinical features and gene expression of many inflammatory cytokines and inflammation/oxidative stress-related proteins [49]. Saresella et al. (2016) showed increased activation of multiple inflammasome complexes and increased intestinal permeability in ASD children and healthy siblings [102]. A systematic review and meta-analysis by Saghazadeh et al. (2019) confirmed the increase of pro-inflammatory cytokines in ASD [103].

As a whole, findings about immune abnormalities in ASD support evidence of an early and ongoing dysfunction in the peripheral immune system and the brain [104]. Furthermore, severe immune alterations are demonstrated also in the non-ASD siblings of ASD patients [105]. Notably, the increase of IL-10—a cytokine with strong anti-inflammatory properties—could be interpreted as a way whereby the immune system tries to counterbalance the inflammation present both in autistic patients and in their non-ASD siblings [105].

Research on neuroinflammation in ASD benefits from the growing body of knowledge concerning the microbiota-gut-brain (MGB) axis, that is the complex multidirectional interactions between central nervous system, gut, gut microbiome, mucosal immune system, enteric nervous system (ENS), autonomic nervous system (ANS, predominantly the vagal branch which directly innervates the gut) [106].

A pivotal role in the MGB axis is played by central processes receiving input from the vagus nerve with vagal afferent information relayed to the hypothalamus via the nucleus tractus solitarius and area postrema in the caudal brainstem [107].

Accumulating evidence reveals the multifaceted features of the disturbances of the MGB-axis in the pathophysiology of brain disorders and its potential mechanisms [108]. According to this perspective, the numerous factors that can influence the microbiota in the *First 1000 Days* (i.e., maternal microbiota, mode of delivery, breastfeeding, nutrition, infections and early antibiotic therapies) should be considered crucial risk/health factors for neurodevelopment [14,109,110].

The ANS has a pivotal role in the regulation of body’s physiology, beginning before birth, and contributing to fetal development by allowing the organism to efficiently cope with endogenous and exogenous stressors [111]. In particular, the vagus nerve is involved in numerous crucial pathways during fetal, perinatal, and postnatal life: the anti-inflammatory cholinergic action [112], the modulation of bioenergetic metabolism through the production of hormones [113], the role of primary afferent pathways from peripheral organs (in particular, from the gut) to the brain are some of the functions of the vagus nerve. The ANS is the most important bottom-up pathway, which informs the brain about the gut milieu influenced by nutrients, immune response, microbial balance, and toxicants. The complex interplay in which the ANS is involved from the early stages of life explains the fundamental role played during the ontogenesis and why “critical windows” of vagus maturation are in close relationship with the physiology of neurodevelopment [114,115]. Therefore, the control of the potential adverse environmental factors that impact on ANS maturation might have crucial effects on health across lifespan.

In the past decade, several studies focused on ANS activity in ASD (reviewed by Kong et al., 2021 [116]), and it is hypothesized that ANS instability and heightened sympathetic response to stressful stimuli might contribute to the high prevalence of psychiatric comorbidities in ASD [117] and the high prevalence of cardiovascular mortality and morbidity in individuals with ASD [118]. The vagal branch of the ANS directly communicates with the ENS. Studies in animal models demonstrated that both the vagus nerve and the ENS can be affected by gut mucosal inflammation as well as by microbial byproducts such as SCFA (short-chain fatty acids) and neurotransmitter-like substances [119], underlying the crucial role of the microbial balance for health.

Within the gut-brain axis, the microbiota behaves like the conductor of the immune-neuroendocrine communication orchestra [120,121,122,123]. Numerous molecular pathways have been suggested to explain the correlation between the microbiota composition and brain wiring in neurodevelopment: direct epigenetic mechanisms by bacterial-derived products on the histone proteins of brain cells [124]; production or alteration of neurotransmitters, including serotonin [125]; dysbiosis-induced breakdown of gut integrity with consequent permeability changes in the enteric barrier and increased transit of substances from the intestinal lumen to the lamina propria [74,126]; production of toxins [127]; activation of immune signaling pathways, with release of cytokines and other inflammatory molecules [101,104,128] including inflammasome activation [102]; direct activation of the vagus nerve [125]; aberrations in fermentation processes or products [129,130].

Numerous studies demonstrate a correlation between the state of unbalanced microbial communities—named “dysbiosis”—and a wide range of diseases [131,132], including neurologic abnormalities, from neurodevelopment disorders to psychiatric conditions [124,133,134] and neurodegenerative diseases [135,136,137,138,139,140]. The link between dysbiosis, ASD, and gastrointestinal (GI) has received growing interest [138,139,140]. Numerous studies demonstrated the difference of the bacterial composition between ASD cases and controls (reviewed by Bundgaard-Nielsen et al. (2020) [141]. Ding et al. (2021) recently confirmed a striking association between ASD and GI symptoms as well as remarkable differences in gut microbiota composition between ASD children and controls [142].

Converging evidence suggests that the gut-brain axis and the numerous mechanisms involved in its function play important roles in the pathogenesis and symptoms in ASD. Deciphering the relationships in this complex system and translating them into clinical practice is a great and difficult challenge that we must face. There is no doubt that this is a crucial issue for the health of autistic people. In fact, the microbiota imbalance affects several pathophysiological processes.

Vitamin deficiencies are among the most frequent metabolic abnormalities related to gut microbial imbalance and are frequent in ASD. B-vitamin deficiencies are of utmost interest for their key role in numerous physiological processes involved in brain energetic metabolism and cell respiration, such as the metabolism of glucose, fatty acids, and amino acids, homocysteine metabolism, metabolism of tryptophan in the kynurenine pathway, synthesis and metabolism of various neurotransmitters and neurohormones [143]. B-vitamins are also involved in the regulation of permeability of the intestinal and blood-brain barriers and have a broad range of immunomodulatory, anti-oxidant, and neuroprotective properties. Their role has been evaluated in a broad variety of neurologic and psychiatric disorders, including bipolar disorder, major depression, schizophrenia, autism, Alzheimer’s, and Parkinson’s diseases [143,144,145]. Dietary sources, gut microbiota, and fermented food rich in probiotic bacteria are the essential sources of B-vitamins [146]. Therefore, dysbiosis and nutritional imbalances might endanger a fundamental system for human health, in particular those related to the nervous system physiology.

In the postmortem frontal cortex, Zhang et al. (2016) demonstrated concentrations of vitamin B12 more than three-fold lower in both autistic and schizophrenic subjects than in age-matched controls [147]. Simultaneous vitamin B6, B9, and B12 deficiencies have been demonstrated in ASD [148]. These findings are consistent with the abnormalities of protein and DNA methylation reported in autistic children [149,150]. Besides the B vitamin deficiencies, Belardo et al. (2019) demonstrated a significant block of cystathionine formation, consequent accumulation of homocysteine, and lower levels of the reduced form of glutathione (GSH), causing a decrease of an essential component of the intracellular reducing machinery, required for the physiologic immune response, detoxification capacity, and redox-sensitive enzyme activity [148]. Glutathione deficiency in autism was first described by Chauhan and Chauhan in 2006 [151]. Deficiencies in B-complex vitamins (B1, B6, B9), in sulfur-containing antioxidants, as well as in other micronutrients, are likely a consequence of the activity of toxic aldehydes, which are generated during the peroxidation of membrane lipids under oxidative stress [152].

Abnormalities in tryptophan pathways are also a relevant metabolic issue, which is reported in ASD and might be linked with dysbiosis. Tryptophan is an essential aromatic amino acid and is one of the pivotal metabolites in microbiota–host crosstalk in health and disease. Levels of this amino acid depend on diet intake and on the composition of gut microbiota. Microbial balance controls the three major tryptophan metabolism pathways leading to serotonin, kynurenine, and indole derivatives [153]. Numerous studies found lower levels of tryptophan in ASD children compared to controls [154,155]. Gevi et al. (2016) demonstrated a large perturbation in the tryptophan metabolic pathway in ASD, with prevalence of the kynurenine pathway and transmutation to xanthurenic acid and quinolinic acid (two catabolites of the kynurenine pathway) [156]. Assuming that the metabolic imbalance documented in urine reflects a similar biochemical imbalance in the CNS, the urinary metabolomic pattern is consistent with enhanced oxidative stress and excitatory neurologic imbalance, fostering the risk of seizure occurrence in 20% of the ASD children in the sample [156]. A further consequence of the preferential metabolism of tryptophan along the main branch of the kynurenine pathway is the relative decrease in the production of serotonin and melatonin in ASD. These findings might contribute to explain abnormalities in behaviors, disturbed circadian rhythms, and eating disorders in ASD [156].

Increased levels of homocysteine in ASD have been reported by several studies [148,157,158,159]. Besides the involvement in neurotransmitters synthesis, homocysteine leads to increased excitatory glutamatergic neurotransmission, which initiates the cascade of calcium imbalance, mitochondrial dysfunction, increased oxidative stress, and progressive neuron dysfunction and degeneration [160]. These mechanisms explain why mild to moderate hyperhomocysteinemia is a risk factor for neurodegenerative diseases [161] and raise concerns about the occurrence of progressive neurodegeneration in ASD. Several studies demonstrate signs of an ongoing neuroinflammatory and neurodegenerative process in different regions of the brain involving microglial activation [99,162,163,164]. In post-mortem ASD brain tissue, Vargas et al. (2005) documented activated microglia and astrocytes in the cerebral cortex, white matter, and notably in cerebellum. They also found a consistent increase in the expression of the proinflammatory chemokine monocyte chemotactic protein-1 (MCP-1) and the correlation between the elevation of MCP-1 and microglial activation in the same area. The authors inferred that the correlation might be explained by the chemotactic recruitment of monocytes/macrophages to areas of neurodegeneration they found in the cerebellum [99]. Lastly, they noticed that these observations are similar to findings in studies of other neurodegenerative diseases in which elevation of MCP-1 is demonstrated, such as human immuno-deficiency virus (HIV) dementia, amyotrophic lateral sclerosis, and multiple sclerosis [99].

Research in this field should be encouraged in order to ascertain the risk of neurodegeneration and engage appropriate prevention strategies toward adulthood. Furthermore, evidence suggests that future therapies might involve modifying neuroglial responses in the brain [99].

Among the products of the gut microbiota and in relation to neuroinflammation, SCFA are worth mentioning, as these participate in manifold processes of utmost interest for neurodevelopment, from the increase of gut permeability to epigenetic modifications in the brain [134]. In particular, butyrate is a small SCFA able to cross the blood-brain-barrier, has anti-inflammatory and neuroprotective effects [165], and its administration in animal models of autism leads to the attenuation of social behavior deficits [166]. Furthermore, butyrate supports mitochondrial function through the oxidative phosphorylation and fatty acid oxidation [167], thus linking the microbiota composition/functions to oxidative stress/mitochondrial function. In fecal samples from ASD children butyrate concentration resulted lower than controls [168]. Butyrate supplementation showed positive effects in lymphoblastoid cell lines derived from children with ASD, enhancing mitochondrial function in the context of physiological stress and/or mitochondrial dysfunction [169].

Mitochondrial abnormalities and oxidative stress in ASD are treated last because it allows us to link mitochondrial dysfunctions to numerous abnormalities that we have considered in the course of the article.

Mitochondria are cell organelles with matrilinear transmission and their own DNA. They are essential for a wide range of functions, such as the production of adenosine triphosphate (ATP) by oxidative phosphorylation, redox metabolism, lipid homeostasis, steroid synthesis, calcium buffering, regulation of apoptosis and metabolic pathways of the urea cycle, porphyrin and amino acid production [170,171]. Mitochondria play a key role from the earliest stage of life, from the first division of the zygote, and participate to the machinery that “oils chromatin dynamics” required for cell proliferation and differentiation during the ontogenesis [172]. Furthermore, these organelles regulate neural stem cell fate decisions and impact neurogenesis both during neurodevelopment and in adult brain [173]. Mitochondria are sensitive stress targets in the placental microenvironment, and play a crucial role in the harmonious maternal–fetal crosstalk [174]. Noteworthy, their dysfunction has been correlated to placental abnormalities in gestational disorders [175].

Mitochondria are key targets of many environmental contaminants and toxicants [176,177]. This issue is particularly compelling in ASD and supports the hypothesis that mitochondria may mediate prenatal environmental influences in ASD [178], which is consistent both with the previously mentioned epigenetic paradigm and with the frequent finding of mitochondrial abnormalities in ASD. A systematic review and meta-analysis found that about 30% of children with ASD exhibited biomarkers consistent with mitochondrial dysfunction [179]; moreover, the function of the electron transport chain (ETC the machinery fueling energy production) in lymphocytes resulted lower than controls in 80% of children with ASD [180], confirming mitochondrial dysfunction. As discussed by Bargiela and Chinnery (2019), mitochondrial dysfunction is associated with neuroinflammation and neurodegenerative disease, however in most instances the mitochondrial dysfunction is acquired rather than due to DNA mutations [181]. Oxidative stress and mitochondrial dysfunction are interconnected and influence each other, since oxidative stress damages mitochondria and dysfunctional mitochondria produce reactive oxygen species (ROS) [182]. This consideration has profound implications in the identification of oxidative stress as a feasible therapeutic target in ASD.

Both increased oxidative stress and imbalance in cell membrane fatty acids composition, a result of free radical attack to polyunsaturated fatty acids, resulting in dysfunctional cell membranes, have been shown in ASD children [183], where some clinical features (in particular, hyperactivity and cognitive development) correlated with the unbalance in lipidomic profile, red blood cells membrane fluidity, and decreased activity of membrane-embedded enzymes. This suggests that the membrane lipidomic profile could be a useful biomarker for personalized therapeutic supplementation [184,185,186]. At the same time, laboratory findings suggest that oxidative stress may lead to a chronic state of peripheral hypoxia in autistic people. If so, a progressive increase of the oxidative stress and a continuous deterioration of health in ASD people are predictable events.

Following the hypothesis that oxidative stress represents a hallmark of metabolic abnormalities in several brain disorders and given that it influences a wide array of molecular pathways, the possible impact of this imbalance was studied in relation to the impairment of chloride homeostasis occurring in the GABAergic system in CNS [187]. This study showed evidence linking the impairment of intracellular chloride concentration with the occurrence of oxidative stress and inflammation in patients with different neurological and neurodevelopmental diseases, such as ASD, epilepsy, schizophrenia, Down’s Syndrome [187]. Moreover, it showed that increased oxidative stress interferes with the shift of GABAergic transmission from excitation to inhibition, a fundamental ontogenetic process under epigenetic control [188]. This observation leads further support to the importance of primary prevention during pregnancy.

The broad array of fuzzy interactions described above shows that biological complexity underlying behavioral characteristics of ASD is very different from a traditional linear model of “a symptom, a biomarker”. Metabolomics gives the chance to get away from the “juniper bush” of ASD [189]. It allows the systematic identification and quantification of the global collection of all metabolites, the metabolome, that are recognizable both in biological fluids and in tissues [190]. Metabolomics identifies metabolic networks shaped by nodes (metabolites) and their interactions (scale-free network models) [191]. In other words, metabolomics describes the individual molecular phenotype, and allows monitoring of its changes over time through a “meaningful web” representing the individual “biochemical fingerprint”. Growing evidence highlights a wide array of applications of metabolomics in the study of neurodevelopmental and neuropsychiatric disorders, such as monitoring of fetal and perinatal programming for risk of neurodevelopmental/neuropsychiatric disorders [192,193,194], diagnosis of neurodevelopmental and psychiatric disorders [195,196], early diagnosis of ASD, risk of regressive ASD [197], correlation between metabolome and clinical phenotype in ASD [198]. The most discriminant metabolites in ASD are involved in amino acid metabolism, antioxidant status, mitochondrial function, and nicotinic acid metabolism [199]. Most of the studies in ASD found abnormalities in microbial metabolites and in intermediate compounds of the Krebs cycle [200,201]. Therefore, metabolomics open very promising perspectives in prevention, diagnosis, therapy, and follow-up in ASD, allowing a personalized approach to unique and evolving needs.

In conclusion, a wide array of interconnected metabolic, immunologic, microbiologic abnormalities is found in ASD people. From the earliest stage of life and across life span, the interconnections between these three systems are always active and are crucial to restore the homeostatic balance for health. In the case adverse events persist for a long time or when they exceed the possibility to restore the balance, a vicious circle is established between inflammation, mitochondrial dysfunction/oxidative stress, and dysbiosis. These three mechanisms, as a whole, form a “Bad Trio”, with progressive imbalances and dysfunctions not limited to the nervous system and a major impact on fetal development [14]. Subsequent to the embryo-fetal period, the greatest impact on neurodevelopment occurs in the first two years of life, i.e., in the period of most active neuroplasticity. After birth, the vicious circle of the “Bad Trio”, if not blocked, continues and undermines the health of ASD people throughout life.

## 4. Much More Than Behavioral Abnormalities in People with ASD

Autistic people show several comorbidities besides neuropsychiatric conditions. A high prevalence of obesity and obesity-related disorders (type 2 diabetes mellitus, hypertension, hyperlipidemia, and non- alcoholic fatty liver disease/nonalcoholic steatohepatitis) have been reported in children [202], which might in many instances be iatrogenic, although the use of psychotropic drugs is probably not the only explanation for these abnormalities. In fact, recent evidence suggests that metabolic syndrome may precede and even exacerbate long-term side-effects of psychiatric medications [203]. Other prevailing comorbidities are allergies and gastrointestinal (GI) diseases, which are predictable, given the wide array of immune imbalances and the intestinal dysbiosis described in ASD [13,204]. Besides dysbiosis, other gut abnormalities of ASD people include, in particular, GI motility and intestinal permeability [205,206]. In a large sample of ASD adults, GI complaints are reported in 21% of patients [207]. Abdominal pain/discomfort, vomiting, reflux, diarrhea, constipation, flatus, and unusually foul-smelling stools are more frequent in ASD than in controls [208,209]. The most comprehensive meta-analysis to date revealed that children with ASD were more than four-fold more likely to develop GI disorders than controls; constipation, diarrhea, and abdominal pain are the most common ailments reported [210]. The wide range of variability of GI symptoms, affecting from 9 to 70% of the ASD patients, is probably explained by differences in the populations studied and by different assessment tools [209]. Moreover, the rare eosinophilic enterocolitis is more frequent in ASD than in the general population [211]. This is a difficult challenge in clinical practice, since this eosinophilic enterocolitis often causes severe pain but not a significant increase in biomarkers of inflammation. Therefore, in case of well-founded clinical suspicion advanced by an expert physician, digestive endoscopy and histological examination including eosinophil count have to be executed to make a diagnosis of eosinophilic enterocolitis. GI symptoms are a crucial issue among health complaints in people with ASD, and have a severe impact both on wellbeing and on behavioral abnormalities [138,204,209]. Pain and discomfort are often underdiagnosed in people with intellectual disability. The different neural integration of somatosensory inputs and difficulties in communication make the risk of underestimation of pain even higher in ASD people [209]. Specific tools for the evaluation of GI symptoms and pain should be systematically included in clinical assessment in ASD, including parallel monitoring of behavioral symptoms [212]. In fact, the behavioral functional analysis methodology requires an accurate evaluation of the events that precede the elicited behaviors, taking into account that the antecedent situation includes any input from the internal as well as the external environment [213]. Therefore, a preliminary medical assessment for the identification and treatment of comorbidities in ASD is not only an inalienable right to health, but also the necessary condition for a correct behavioral analysis, in order to provide competent educational support and avoid the risk of incorrect psychotropic treatments. For this reason, assessment in ASD requires a multidisciplinary team and an interdisciplinary approach.

Epilepsy is another frequent comorbidity in ASD [214,215,216]. The wide array of immunologic abnormalities in ASD suggests a possible role of neuroinflammation in the pathogenesis of epilepsy [217]. Epilepsy has an enormous impact on health and quality of life of ASD people. The high frequency of drug resistance and the elevated risk of drug adverse side effects [218] suggest investigating the impact of systemic inflammation on the etiopathogenesis and progression of epilepsy in ASD [219]. Moreover, the link between epilepsy and oxidative stress [187] should not be overlooked, confirming the multiple and evolving effects of oxidative stress on the health of autistic people.

Hopefully, a better understanding of the gut-brain axis and of systemic neuroinflammation will provide insights into the onset and progression of frequent neuropsychiatric disorders in ASD [220] and into regressive events that might be related to autoimmunity events occurring in CNS [221]. In particular, the potential co-occurrence with Pediatric Acute-Onset Neuropsychiatric Syndrome (PANS) deserves to be explored in regressive events that sometimes occur in the life of autistic people. The data mining approach appears to hold promise for research in this direction [222].

## 5. Lessons from Biological Research and Suggestions for Clinical Practice

The paradigm shift from genetics to epigenetics highlights the priority of primary prevention during pregnancy [14]. Fetal neurodevelopment is conditioned by many interacting factors that influence uterine environment. A wide range of damaging events occurring during pregnancy may initiate the three aforesaid major interacting/overlapping pathways (dysbiosis, mitochondrial impairment/oxidative stress and immune activation (MIA)) that impact epigenetic machinery during ontogenesis and affect the brain neural wiring. The matrilinear transmission of both microbiota and mitochondria [223,224] further strengthens the need for effective women’s health programs.

Numerous studies have assessed the characteristics and the frequency of potential environmental factors in pregnancy which increase the risk of ASD in the offspring [50,63,83]. The discovery of synergistic effects of different endogenous and exogenous factors well reveals the occurrence of biologic interactions. The separate assessment of each single exposure, as it is traditionally carried out, appears to be an oversimplification compared to the complexity of biologic systems. In this regard, the application of machine learning systems is very promising for the development of predictive models to track the risk profiles in the lifelong neuropsychiatric trajectory [83].

Many risks and protective factors have been linked to the onset of ASD in the offspring, providing suggestions for clinical practice. Numerous studies focusing on the risk of ASD in the offspring demonstrate the relevance of carefully designed maternal diet, including nutraceutical supplementation [50,225], the prevention and treatment of metabolic abnormalities [226], avoidance of some toxicant exposure [227]. However, there is a striking discrepancy between the plethoric literature in this field and the scarce transfer of knowledge to clinical practice. Mothers of autistic children usually report that only general advice is provided about diet, nutritional state, nutraceutical supplementation, care for gut microbiota balance, lifestyle. Mothers are often not told that alcohol in pregnancy must be absolutely avoided, even in very modest quantities. In this regard, it should be emphasized that it is very difficult to obtain information about alcohol consumption during pregnancy [228]. Foster and adopted children have a higher prevalence in fetal alcohol spectrum disorder (FASD) [229], a leading cause of neurodevelopmental disorders which pose a risk of ASD and ADHD respectively 2 and 15 times higher in comparison with the general population [230]. A large survey addressed to Fellows of the American College of Obstetricians and Gynecologists investigated knowledge, opinions, and practice regarding alcohol-related care [231]. Eight hundred obstetrician-gynecologists were selected and 48% returned the survey. Most of respondents asked pregnant patients about alcohol use only during patients’ initial visit, whereas about 10% asked the same questions also during subsequent visits. One-third of professionals did not indicate that occasional alcohol consumption is not safe during any period of pregnancy. When asked which validated alcohol risk screening tool they most commonly use with pregnant patients, about 60% said they use no tool [231]. The survey of knowledge about FASD and alcohol screening practices used previously with obstetricians by Anderson et al. (2010) was adapted for use with nurses and midwives by Chiodo et al., (2019): more than 40% of the respondents believed drinking alcohol is safe during at least one trimester of pregnancy [232]. In order to favor awareness and best practices, some countries provide health professionals with educational resources about prevention of prenatal alcohol exposure and fetal alcohol spectrum disorder and assess changes in their knowledge over time [233]. It is to be hoped that this is an example that will be followed. An early diagnosis of prenatal alcohol exposure offers a crucial temporal “window” of intervention through the supplementation of mother’s diet with protective and antioxidant substances in addition to supportive psychological therapies [234].

Maternal fetal stress is another important risk factor, with numerous and complex effects on neurodevelopment [235], but very little attention has been paid to this aspect. It appears that the slogan “pregnancy is not a disease” has been instrumentally twisted in a society always in a rush. Maternal stress is not cared for even during the second pregnancy in families that already have an autistic child, in case the second pregnancy occurs while mothers are facing the stressful diagnostic route for the first autistic child. It is recommended that more attention should be paid, both in research and in clinical practice, to maternal stress, as well as to specific strategies aimed at primary specific prevention for autistic mothers. In fact, the scarcity of data published on this topic suggests the need for increased specific support for autistic women during their motherhood [236].

The second issue proposed in this study concerns the application of the systemic perspective in clinical practice. A large population-based study by Hirvikoski et al. (2016) [237] confirmed the increased mortality in ASD showed by previous clinical and population-based mortality studies [118,238]. Individuals with ASD had a 2.56 fold increased odds of mortality compared with matched general population controls. Mortality was increased in both low-functioning and high-functioning ASD, as well as in both genders. The risk was higher in low-functioning ASD (OR 5.78) and it was particularly high for females with low-functioning ASD (OR 8.52) [237]. The authors found that increased mortality in ASD was not limited to specific causes of death, but was elevated for all analyzed categories, indicating that ASD accounts for substantial health loss across the lifespan. These findings confirmed the results of a previous systematic review aimed at understanding the epidemiological and global burden of ASD [239].

Adopting a systemic perspective in place of the behavioral-centered approach, health needs of autistic people appear much more complex and require a wide array of professional expertise for assessment and treatment, in the medical as well as in the educational field. Medical semeiotics is different in individuals with ASD compared to general population. Differences in somatosensory integration may influence pain threshold and therefore change perceptions and awareness of somatic disturbs. Moreover, impaired communication skills often make it difficult to inform caregivers and doctors about pain and organic ailment [209]. As a consequence, the manifestation of discomfort and pain may be unusual and emerge through challenging and disruptive behaviors. Therefore, medical expertise, in particular, by pediatricians and family physicians, is essential to ascertain possible organic ailment underlying behavioral abnormalities.

If the observation of behavioral abnormalities in ASD lacks the biological dimension, there would be high risk of ascribing by default any disruptive and challenging behavior to autism, with consequent risk of incorrect use of psychotropic drugs. The high rate of psychotropic polypharmacy in ASD should be a matter of reflection [240]. Since challenging behaviors are often indications for drug treatment and institutionalization, the interdisciplinary team’s experience is crucial for making the right decisions [241].

The multidimensional perspective is critical in early observation by pediatricians. The frequency of persistent crying (i.e., excessive crying with higher age at onset and longer duration compared to infant colic, according to Rome IV criteria [242]) was significantly higher in ASD compared to control infants (32% vs. 9%, *p* < 0.001), with relative risk 4.40 [243]. The authors assume that persistent crying might be an early symptom of ASD, suggesting timely behavioral screening by pediatricians [243]. Furthermore, early detection of behavioral abnormalities and timely intervention lead to better clinical outcomes [244]. Thus, early screening is critical. On the other hand, knowledge concerning the aforesaid early and complex biological imbalance in ASD calls for a multidimensional evaluation of the pathogenesis of persistent crying in infants and suggests interventions to restore physiologic balance as soon as possible. It is plausible that early and appropriate biological intervention may have positive impact on the risk of brain disorders. Therefore, further studies addressed to fussy babies are warranted. The abnormalities of intestinal barrier in ASD [206], the activation of the vagus nerve [112,245], and the impact of dysbiosis on the blood-brain-barrier [246,247] make early dysbiosis particularly concerning. According to a multidimensional perspective, the early recovery of metabolic balance should be combined to early evidence-based educational support: the combination of these two interventions should produce the best results for a favorable outcome in high-risk infants [248]. Research in this field should be encouraged.

The issue of early interventions is in many ways interlaced with the heterogeneous developmental pathways of ASD children. In this regard, promising insights are expected from metabolomics. The study of the “bloomers” trajectory (i.e., the group of children that evidenced remarkable developmental changes over time [249]) and insights about the mechanisms underlying the “blooming” should be a priority in research and clinical practice. Moreover, the “multiple-hits” frailty trajectory in neurodevelopment [60] calls for the early detection of any biological abnormality which potentially disturbs the brain wiring, in order to restore the best balance as soon as possible, ideally in the period of maximum neuroplasticity. Metabolomics seems to be able to unearth the complex imbalances underlying the ASD phenotype and will probably be a pivotal clinical tool in the next few years. [198,199]. Waiting for metabolomics in clinical routine, the aforesaid frequent and serious health needs of autistic people call for urgent improvement of medical care. After the diagnosis of ASD, intervention should ideally start with a careful medical assessment. The involvement of the gut–brain axis, entailing dysbiosis and increased intestinal permeability [206], and the abnormal lipid composition of cell membranes may be diagnosed by evaluation of fecal calprotectin [250,251], zonulin [252,253], analysis of the microbiota and of some fecal microbial metabolites (mainly, lactate, propionic acid, and butyrate), and by assessing the erythrocyte lipidomic profile [184,185].

In the gut-brain axis framework, diet is crucial. Frequent eating disorders [254] and the use of edible reinforcers in educational intervention [255] contribute to nutritional imbalances, to the risk of metabolic syndrome and of increased oxidative stress [202,256,257]. Besides the energy intake, diet impacts immune function [258], microbiota composition [259,260], and lipid cell membrane profile [261]. In other words, diet impacts the main disturbances demonstrated in ASD. Therefore, nutritional experts should be included in the multidisciplinary panel for healthcare model in ASD, in order to provide individualized dietetic plans.

**Figure 1:**
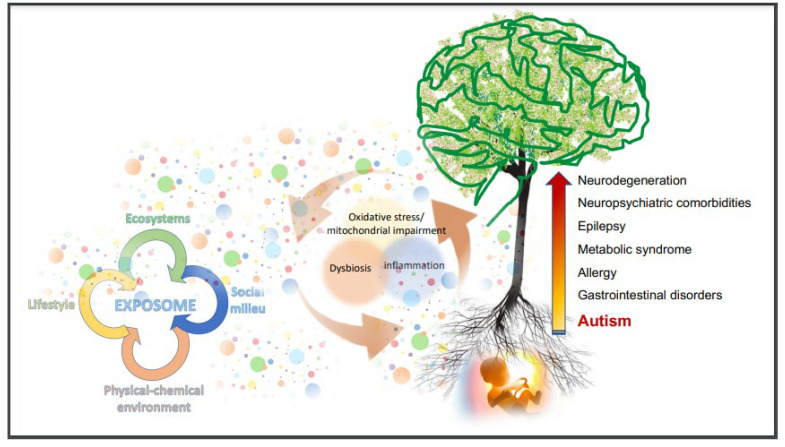
The recent upward trajectory in prevalence and the multifaceted phenotype in ASD suggest that autism is in most cases the result of an early perturbation of ontogenetic pathways, with different age-related outcomes, in line with the DOHaD Theory [34,37,38]. The early start of a vicious circle between inflammation, dysbiosis, and oxidative stress/mitochondrial impairment (the “bad trio”) launches a multi-step frailty trajectory, and inserts the major comorbidities in ASD within the pathogenetic framework. From the womb to adulthood, the nervous system might be progressively involved, from inflammatory to degenerative processes. The circle of the “bad trio” is in relationship with the environment, in the broad sense of the “exposome” (the colored dots), that is the sum of all the factors our body is exposed to. Both risks and opportunities for human health come from the exposome, and major impact occurs through the action of the bad trio. The substantial loss of health across the lifespan and the increased mortality in ASD encourage best efforts aimed at “improving the soil around the tree”.

A very rich literature supports the notion that oxidative stress occurs in ASD as well as in several, if not all, brain diseases and disorders [187]. Sources of oxidative stress are manifold and include inflammation, gut dysbiosis, xenobiotic and drug metabolism, maternal alcohol abuse, dysfunctional mitochondria. Oxidative stress, in turn, is the main cause of the altered membrane lipid composition, impacting on cell function and on cell-to-cell communication, and affects protein and DNA structure, chromatin modification, mitochondrial activity, immune function, and gut microbiota, often in a self-sustaining loop. Therefore, dietetic interventions should include personalized supplementation with antioxidants and essential fatty acids, and should be aimed at replenishing those micronutrients that are depleted by the direct or indirect action of oxygen radicals [152,155]. The substantial health loss across the lifespan of ASD people [237,239] suggests that this knowledge should be urgently transferred into clinical practice through the establishment of an interdisciplinary model for clinical cooperation [14,262,263]. Figure 1 summarizes in one image the dynamic and systemic perspectives embracing diagnostic criteria and care in ASD.

## 6. Discussion

This review examines the contribution that biologic research could provide to the updating of the healthcare system, in order to meet needs of autistic people of today and tomorrow, from the womb to adulthood.

A recent systematic review and meta-analysis examined age at ASD diagnosis: the subgroup analysis for studies that only included children younger than 10 years found a mean age at diagnosis of 43 months with a range of 31–75 months [264], which means that most of the time clinical diagnosis is still formulated today when best opportunities for neurodevelopment have expired. Therefore, parallel to the enhancement of tools and skills aimed at early diagnosis and psycho-educational intervention, effective health systems should do their best for preventive care in the *First 1000 Days*.

Here, we have presented the reasons why the highest level of well-being throughout life in ASD may be warranted by an integrated model that includes expert and comprehensive medical interventions besides psycho-educational-social support.

The undeniable biological dimension in ASD is evidenced by a broad array of biochemical, immunological, and microbiological imbalances, and data about mortality and morbidity [118,237,238]. Knowledge calls for a change, both in research and clinical practice. Current evidence in ASD is almost entirely based on clinical trials that limit assessments to behavioral characteristics and do not stratify study samples using laboratory biomarkers or standardized evaluation of organic symptoms. The stratification by means of biomarkers was not even considered in meta-analyses concerning biological topics, such as dietary interventions [265]. This lack conflicts with the expected coherence between the phenomenon and the methodology used to study it, suggesting that epistemological and ontological issues should be prioritized to better understand autistic heterogeneity [266].

Knowledge and awareness of the biological bases of ASD increase day by day. A striking number of publications have demonstrated abnormalities in peripheral biomarkers in numerous psychiatric conditions and brain disorders besides ASD [187,267,268,269]. Enlarging the gaze, we find strong convergence between some major neuropsychiatric conditions that are behaviorally defined through DSM-observation criteria (e.g., autism, depression and schizophrenia), and some neurological diseases (e.g., Parkinson’s and Alzheimer’s disease) [270], where a wide array of interplaying pathways involving four major areas (i.e., immune dysregulation, oxidative stress/mitochondrial dysfunction, dysbiosis, and environmental exposures (in the broader sense of “exposome”)) has been recognized [12,14,178]. From early steps in utero to brain functioning in adulthood, converging evidence underscores the complex and inalienable brain–body connection within the bio-psycho-social framework for human health and wellbeing. Hence, biological evidence of brain-body connections integrates the traditional diagnostic approach in psychiatry, which is driven by the observation and description of behaviors [270]. ASD is paradigmatic for the need of a multidimensional integration, since an exclusively behavioral assessment appears simplistic with respect to the broad array of demonstrated biological abnormalities.

The bio-psycho-social model’s sense, in the words of its founder Engel, is that “all three levels, biological, psychological, and social, must be taken into account in every health care task” [15,16]. This model provides integrated, holistic explanations for the development, progression, and treatment of diseases and is equipped to explain their increasing complexity. Biological evidence in ASD forces the overcoming of the dualistic conception of body and mind and well matches Engel’s suggestion to build bridges between the two realms [271].

Within the interconnected bio-psycho-social framework, one of the most intriguing challenges in ASD deserves a mention: the enhancement of “thinking different” and the awareness that psycho-neurodiversity and disease are not the same [5]. Establishing the boundary between neurodevelopmental disorders and value of neurodiversity is essential. In fact, the former requires best efforts to prevent/minimize an ailment, while the latter calls for respect and enhancement. The coexistence of all these aspects should be the basis for an effective cultural both scientific and social—alliance to bring about a change.

Engel’s paradigm is still valid [272] and new theoretical bases coming from complex systems mathematics support its effective clinical application, providing tools able to describe problems of high complexity by the reproduction of the dynamical interaction of multiple simultaneous factors [273]. The wide array of topics and findings in ASD and the lack of evidence for single biological hallmarks might be confusing and misleading. The perception that biologic research in ASD is an inconclusive Penelope’s canvas, may make us skeptics and hesitant. Artificial intelligence shows a promising and reassuring way to leave the “juniper bush” [189], since it is able to integrate omics data to medical records in ASD [199,274], that is to say, it accepts complexity and helps us to understand it.

**Figure 2:**
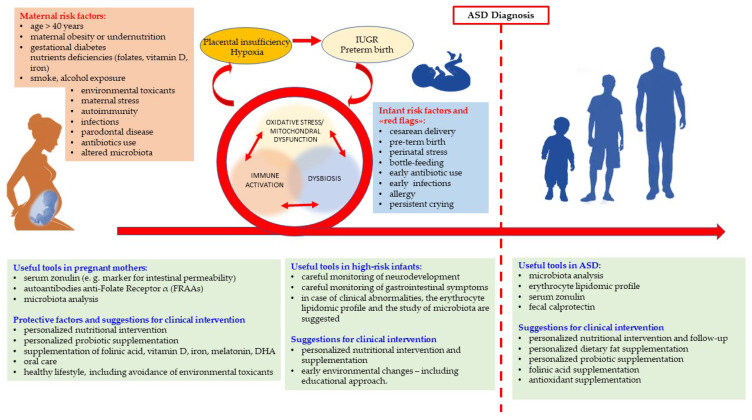
Risk factors for neurodevelopment and suggestions for clinical practice [14,50,60,83,275,276,277,278,279,280,281,282,283,284]. The picture shows some of the most relevant risk factors for ASD. We mention only maternal risk factors, however also some paternal characteristic (in particular, age > 40) are a risk factor for ASD. During pregnancy, a broad array of interconnecting pathways link each factor to the others and in a more or less direct way could contribute to the vicious circle of the “Bad Trio”. In some cases, the link is more evident: antibiotic use favors dysbiosis; infections in pregnancy cause immune activation. Mechanisms involving other risk factors might be not so straightforward—e.g., mother’s health status may or may not affect low-grade inflammation in numerous metabolic disorders, such as obesity and diabetes. Nutrient deficiencies may be prevented or corrected through specific supplementation, provided they are diagnosed in time. The relevance of gut permeability and microbial balance within the immuno-neuro-endocrine system underscores the usefulness of some laboratory assays, such as the evaluation of maternal serum zonulin and the microbiota analysis. As far as the role of the microbial balance, evidence concerning the relationship between maternal oral dysbiosis and the risk of ASD in offspring underscores that the gut microbiota is not the only one to pay attention to. A further important investigation extensively discussed in the text concerns the titration of anti-FR α autoantibodies. Intra-uterine growth retardation (IUGR) and pre-term birth are the most frequent outcomes of the abovementioned multi-etiological placental insufficiency. They are the result of a previous frailty trajectory and themselves are the premise of further risks, involving, once again, the “bad trio”. In fact, there is a correlation between IUGR, pre-term birth, increased oxidative stress, susceptibility to infections, and consequent early antibiotic use. The careful monitoring of the neurodevelopmental trajectory of high-risk children should go hand-in-hand with the monitoring of biological abnormalities and gastrointestinal symptoms. Persistent crying (discussed in the text) should be considered a red flag, indicating early ANS abnormalities, that suggest investigating through the study of microbiota and of erythrocyte lipidomic analysis. Perspective studies are required to ascertain if the early and tailored restoration of the biological balance has positive impact on neurodevelopment of high-risk infants. Similar considerations concern the biological abnormalities in children and adults with ASD, that are represented to the right of the red dashed-line. Personalized supplementation and nutritional adjustments suggested in the green box might tune the metabolic, immunologic, and microbiologic imbalances and favorably impact both neurodevelopment and comorbidities.

The transfer of the theoretical construct of the bio-psycho-social paradigm into concrete and effective clinical practice requires a rethinking of current training courses for doctors, psychologists, educators, and therapists. Otherwise, the multidimensional construct is likely to be a rhetorical slogan and just an illusion with no positive effects for people lives. Current professional training courses are strongly unbalanced and deal almost exclusively with psychosocial matters, leading researchers and clinicians to fix their gaze on what they are accustomed to seeing. As a consequence, change seems unlikely, both in research and in clinical practice. Therefore, a cultural shift and the rethinking of training models is likely to be the starting point to look at and answer the unmet needs in ASD. Figure 2 summarizes the most relevant risk factors in ASD, as well as the interventions that could reduce or prevent their effects.

## 7. Conclusions

People in the autism spectrum are presenting us with a unique challenge involving every aspect of human action, from the scientific area, to social, economic, and ethical fields. The wide variability in presentation and the extremely diversified needs of ASD people require personalized answers, within the harmonious framework of bio-psycho-social wellbeing.

The topics we have gathered in this study suggest that the answers we get from research largely depend on which questions we ask and what tools we rely on. There are still unexplored fields in healthcare that should be urgently addressed, e.g., the reasons of health loss and of different life expectancy for people in the spectrum disorders compared to the general population.

The current hardships reported by autistic people and their families and the expected worsening of their difficulties and social burden in the next years due to the increased prevalence of ASD underscore the need for timely and concrete solutions. Current knowledge allows us to improve ASD people’s care right now and to imagine different life trajectories and outcomes for them in the future.

Autistic people and their families cannot achieve these results by themselves. They need to be supported by a multidimensional approach based on an open, dynamic, multi- and inter-disciplinary collaboration, both in research and in clinical practice.

## Data Availability

Not applicable.
